# Supplementary material for: Exome sequencing early in outpatient evaluation in NCGENES 2: Changing the course of the diagnostic odyssey?
Source: HGG Adv. 2026 Apr 3;7(3):100605. doi: 10.1016/j.xhgg.2026.100605 (PMC13137186; doi:10.1016/j.xhgg.2026.100605)
Supplement: Document S1. Supplemental note [file mmc1.pdf]

HGGA, Volume 7

Supplemental information

Exome sequencing early in outpatient evaluation in NCGENES 2: Changing the course of the diagnostic odyssey?

Tamara S. Roman, Shannon M. Gray, Tam P. Sneddon, Ann Katherine M. Foreman, Kristy Lee, Cynthia M. Powell, Karen E. Weck, Jonathan S. Berg, and Bradford C. Powell

Supplemental Note: Case Reports

15151931CSER: an 8-year-old male referred with hearing loss, mild intellectual disability and seizure disorders. A heterozygous *CSNK2B* NM_001320.7:c.303C>G NP_001311.3:p.(Tyr101Ter) variant was identified. At initial molecular analysis, pathogenic *CSNK2B* variation was not well characterized in the literature, limited to a few previous case reports.[1–3] The phenotypes of that series of patients included developmental delay, mild facial dysmorphism, and epilepsy. Since our initial analysis, pathogenic *CSNK2B* variants have since been reported in multiple studies of individuals with intellectual disability with or without epilepsy,[4,5] and *CSNK2B* is classified as definitively associated with autosomal dominant Poirier-Bienvenu neurodevelopmental syndrome [MIM: 618732] by ClinGen.[6]

15197420CSER: a 4-year-old male enrolled with a primary indication of unspecified hearing loss, also noted to have growth and developmental delays. ES revealed a variant in *GATA3*: NM_001002295.2:c.1256C>T NP_001002295.1:p.(Thr419Met), classified as a Variant of Uncertain Significance (VUS) for *GATA3*-associated hypoparathyroidism, sensorineural deafness, and renal dysplasia [MIM: 146255]. Despite a relatively high population allele frequency (0.003758 in the admixed American population in gnomAD v2.1.1), we considered the reported phenotypic variability and age-dependent manifestations of this condition and reported this variant as a VUS with possible contribution to the patient's phenotype. Parental testing was performed since *de novo* status would be sufficient for reclassification, but the variant was shown to be paternally inherited. The father had a reported history of speech delay but no documented hearing loss and normal renal ultrasound. Endocrine

evaluation of the father was recommended. Considering the possibility of variability of expression and the incomplete phenotypic information available for the father, we could not exclude the *GATA3* variant as causative, so the case-level result was inconclusive due to variant uncertainty.

15254564CSER: a 8-year-old male with autism spectrum disorder, seizures and spastic tetraplegic cerebral palsy. Five variants were reported after exome variant analysis: 3 VUS in *TECTA*, *TRIO* and *ZMYND11* and 2 Likely Pathogenic (LP) variants in *ATL1* and *DHPS*. *ATL1* has been associated with adult-onset hereditary sensory neuropathy type 1D [MIM: 613708] and Spastic paraplegia 3A [MIM: 182600], both as autosomal dominant conditions. With subsequent clinical evaluation, the patient was noted to have more pronounced facial asymmetry, developmental delay, and finger pads. The evaluating clinician considered the *ATL1* finding as probable positive with respect to the participant's spasticity but not explanatory of the patients' other phenotypes.

15316850CSER: a 15-year-old female with a personal and family history (in her sister) of idiopathic osteoporosis. She was found to have a heterozygous *COL1A2* NM_000089.4:c.964G>A NP_000080.2:p.(Gly322Ser) LP variant which was returned as a probable positive case-level result for *COL1A2*-related Osteogenesis Imperfecta. Testing of the participant's sister detected the same *COL1A2* variant. Phenotypically, these patients are most consistent with mild Osteogenesis Imperfecta.

15346997CSER: a 9-month-old referred with short stature, bilateral blurry vision, and encephalopathy. Variants within genes on PanelApp Neurodevelopmental Disorders and Ophthalmological Disorders lists included four classified as VUS in

different genes with possible relation to the patient's phenotype (Supplemental Table 1). Further discussion with the enrolling clinician and additional phenotype information in the study participant prompted a request for analysis of genes associated with Ehlers-Danlos syndromes. This re-analysis identified a VUS in *COL6A2,* in which pathogenic variants have been associated with varying degrees of myopathy and contractures (Bethlem myopathy 1B [MIM: 620725] and Ullrich congenital muscular dystrophy 1B [MIM:620727]). This variant, NM_001849.4:c.139G>A NP_001840.3:p.(Val47Met), was reported as possibly related to the phenotype in this inconclusive case.

15420762CSER: a 12-year-old female enrolled with thin upper lip vermilion, bulbous nose, upslanted palpebral fissure, specific learning disability, growth hormone deficiency, and obesity. Variants identified by exome sequencing were filtered using the PanelApp Neurodevelopment Disorders gene list. A separate obesity gene panel conducted as part of this participant's usual clinical care identified two *MC4R* variants associated with autosomal dominant childhood-onset obesity: NM_005912.3:c.105C>A NP_005903.2:p.(Tyr35Ter) and NM_005912.3:c.110A>T NP_005903.2:p.(Asp37Val). These variants were reportedly *in cis.* However, because *MC4R* is not included on the PanelApp Neurodevelopment Disorders gene list, these two variants were not reviewed as part of the exome molecular analysis process. Of note, *MC4R* is included on the PanelApp Severe Early-onset Obesity gene list. With respect to the participant's presentation, this result is classified inconclusive, since the *MC4R* findings may only explain the patient's obesity, while other aspects of her phenotype remain unexplained.

15504779CSER: a 3-year-old male with macrocephaly (> 97th percentile), autism spectrum disorder and feeding difficulties. Sequencing detected a heterozygous *PTEN*

NM_000314.8:c.752G>A NP_000305.3:p.(Gly251Asp) VUS. While this variant has been reported previously in the somatic context in a papillary tumor of the pineal region[7], to our knowledge it has not been reported in affected individuals with *PTEN* hamartoma tumor syndrome [MIM: 158350]. We ultimately classified this *PTEN* variant as a VUS due to insufficient phenotypic data available for this study participant, as well as the lack of evidence of a deleterious fitness score in an in vitro functional assay.[8] Thus, the case-level result was deemed inconclusive.

15600677CSER: a 4-year-old female enrolled with developmental delay, hypotonia, and dysmorphic facial features. Sequencing identified two heterozygous missense variants, both reported as VUS: *DAG1* NM_004393.6:c.2194C>A NP_004384.5:p.(Pro732Thr) and *SOX5* NM_006940.6:c.1673G>A NP_008871.3:p.(Arg558His). Case-level results were reported as inconclusive, but the enrolling clinician considered *SOX5*-related Lamb-Shaffer syndrome [MIM: 616803] to be a reasonable fit for the participant's presentation. The parents of the participant consented to targeted sequencing which established the *SOX5* variant to be *de novo*; this provided additional evidence to upgrade the *SOX5* variant to LP, ultimately changing the case-level classification from inconclusive to probable positive.

15640652CSER: a 14-year-old female referred because of osteogenesis imperfecta. She was noted at enrollment to have maxillary lateral incisor microdontia. We identified a heterozygous *WNT10A* NM_025216.3:c.321C>A NP_079492.2:p.(Cys107Ter) pathogenic variant, known to be associated with a spectrum of phenotypes, ranging from isolated oligodontia to complex forms of autosomal recessive ectodermal dysplasia. This finding is a likely explanation for the

patient’s microdontia. However, since the primary indication for testing was a history of recurrent fractures of the long bones, a phenotype not associated with loss-of-function *WNT10A* variants, the case-level interpretation was considered inconclusive with respect to the participant’s reason for presentation.

15762386CSER: a 2-year-old male referred with microcephaly, low weight for age, and developmental delay. Three variants of potential relevance (Supplemental Table 1) were identified through filtering for genes in PanelApp Severe Microcephaly and Neurodevelopmental Disorders gene lists, with none of these results considered sufficient to definitively explain the phenotype. One of these findings was a heterozygous variant in *IFIH1*: NM_022168.4:c.2759T>C NP_071451.2:p.(Ile920Thr) classified as a VUS for Aicardi-Goutieres syndrome [MIM: 615846]. Additional manual review of variants outside of the designated gene lists identified two heterozygous missense variants in *TELO2*: NM_016111.4:c.392G>A NP_057195.2:p.(Gly131Asp), classified as a VUS, and NM_016111.4:c.1100G>T NP_057195.2:p.(Cys367Phe), classified as LP for You-Hoover-Fong syndrome [MIM: 616954] (*TELO2* has since been added to multiple PanelApp lists, including the Neurodevelopmental Disorders list). The *IFIH1* variant was maternally inherited; however, given literature evidence of incomplete penetrance in Aicardi-Goutieres syndrome, the presence of this variant in an unaffected parent was not sufficient to reclassify it as Likely Benign (LB). Familial testing also showed the two *TELO2* variants to have been inherited from separate parents (*in trans*). Upon reanalysis in 2023, there were case reports several affected individuals harboring the c.392G>A, p.(Gly131Asp) variant in homozygosity or in *trans* with a second *TELO2* variant,[9,10] DECIPHER[11] (IDs: 290986, 402567, 414901). With the addition of further

information from the literature, application of the PM3 criterion and the clinician's confirmation of concordance of the participant's phenotype of Yoo-Hoover-Fong syndrome, the *TELO2* c.392G>A variant was reclassified as LP and the final case-level classification was probable positive.

15846862CSER: a 1-year-old female enrolled with global developmental delay, hypotonia, oculomotor apraxis and molar tooth sign on MRI. Variants were filtered using the PanelApp Cerebellar Hypoplasia, Neurodevelopmental Disorders, and Ophthalmological Disorders gene lists (a PanelApp gene list specific to Joubert syndrome was not available). No reportable variants were identified. A manual literature search for genes linked to autosomal recessive Joubert syndrome was conducted, which identified a reported association with *B9D1*.[12,13] Variants in this gene were analyzed separately, as it was not included on any of the selected PanelApp lists at the time of analysis. The participant was heterozygous for *B9D1* NM_015681.6:c.341G>A NP_056496.1:p.(Arg114Gln) and this variant was reported as LP for Joubert syndrome 27 [MIM: 617120]. This variant is predicted to change the last nucleotide of exon 4 (NM_015681.6) and had been previously reported in a patient with a clinical diagnosis of Joubert syndrome.[13] Splicing studies on that previously-reported patient's blood showed out-of-frame skipping of exon 4.[13] However, this participant's overall case-level classification was inconclusive due to insufficient zygosity.

SUPPLEMENTAL FILE WEB RESOURCES

Online Mendelian Inheritance in Man (OMIM): https://omim.org)

SUPPLEMENTAL FILE REFERENCES

1. Poirier, K., Hubert, L., Viot, G., Rio, M., Billuart, P., Besmond, C., and Bienvenu, T. (2017). *CSNK2B* splice site mutations in patients cause intellectual disability with or without myoclonic epilepsy. Human Mutation *38*, 932–941. https://doi.org/10.1002/humu.23270.

2. Sakaguchi, Y., Uehara, T., Suzuki, H., Kosaki, K., and Takenouchi, T. (2017). Truncating mutation in *CSNK2B* and myoclonic epilepsy. Human Mutation *38*, 1611–1612. https://doi.org/10.1002/humu.23307.

3. Nakashima, M., Tohyama, J., Nakagawa, E., Watanabe, Y., Siew, C.G., Kwong, C.S., Yamoto, K., Hiraide, T., Fukuda, T., Kaname, T., et al. (2019). Identification of de novo CSNK2A1 and CSNK2B variants in cases of global developmental delay with seizures. J Hum Genet *64*, 313–322. https://doi.org/10.1038/s10038-018-0559-z.

4. Ernst, M.E., Baugh, E.H., Thomas, A., Bier, L., Lippa, N., Stong, N., Mulhern, M.S., Kushary, S., Akman, C.I., Heinzen, E.L., et al. (2021). *CSNK2B* : A broad spectrum of neurodevelopmental disability and epilepsy severity. Epilepsia *62*. https://doi.org/10.1111/epi.16931.

5. Yang, Q., Zhang, Q., Yi, S., Qin, Z., Shen, F., Ou, S., Luo, J., and He, S. (2022). De Novo CSNK2B Mutations in Five Cases of Poirier–Bienvenu Neurodevelopmental Syndrome. Front. Neurol. *13*, 811092. https://doi.org/10.3389/fneur.2022.811092.

6. Rehm, H.L., Berg, J.S., Brooks, L.D., Bustamante, C.D., Evans, J.P., Landrum, M.J., Ledbetter, D.H., Maglott, D.R., Martin, C.L., Nussbaum, R.L., et al. (2015). ClinGen — The Clinical Genome Resource. N Engl J Med *372*, 2235–2242. https://doi.org/10.1056/NEJMsr1406261.

7. Goschzik, T., Gessi, M., Denkhaus, D., and Pietsch, T. (2014). *PTEN* Mutations and Activation of the PI3K/Akt/mTOR Signaling Pathway in Papillary Tumors of the Pineal Region. J Neuropathol Exp Neurol *73*, 747–751. https://doi.org/10.1097/NEN.0000000000000093.

8. Mighell, T.L., Evans-Dutson, S., and O'Roak, B.J. (2018). A Saturation Mutagenesis Approach to Understanding PTEN Lipid Phosphatase Activity and Genotype-Phenotype Relationships. The American Journal of Human Genetics *102*, 943–955. https://doi.org/10.1016/j.ajhg.2018.03.018.

9. Albokhari, D., Pritchard, A.B., Beil, A., Muss, C., Bupp, C., Grange, D.K., Delplancq, G., Heeley, J., Zuteck, M., Morrow, M.M., et al. (2023). *TELO2* -related syndrome (YOU-HOOVER-FONG syndrome): Description of 14 new affected individuals and review of the literature. American J of Med Genetics Pt A *191*, 1261–1272. https://doi.org/10.1002/ajmg.a.63142.

10. Zhao, Y., Han, Y., Li, N., Fu, W., Luo, G., Tan, Y., and Qian, X. (2023). Novel compound heterozygous mutations in *TELO2* in an infant with You-Hoover-Fong syndrome: A case report and literature review. Open Life Sciences *18*, 20220602. https://doi.org/10.1515/biol-2022-0602.

11. Firth, H.V., Richards, S.M., Bevan, A.P., Clayton, S., Corpas, M., Rajan, D., Vooren, S.V., Moreau, Y., Pettett, R.M., and Carter, N.P. (2009). DECIPHER: Database of Chromosomal Imbalance and Phenotype in Humans Using Ensembl Resources. The American Journal of Human Genetics *84*, 524–533. https://doi.org/10.1016/j.ajhg.2009.03.010.

12. Romani, M., Micalizzi, A., Kraoua, I., Dotti, M.T., Cavallin, M., Sztriha, L., Ruta, R., Mancini, F., Mazza, T., Castellana, S., et al. (2014). Mutations in B9D1 and MKS1 cause mild Joubert syndrome: expanding the genetic overlap with the lethal ciliopathy Meckel syndrome. Orphanet J Rare Dis *9*, 72. https://doi.org/10.1186/1750-1172-9-72.

13. Katiyar, D., Anderson, N., Bommireddipalli, S., Bournazos, A., Cooper, S., and Goel, H. (2020). Two novel B9D1 variants causing Joubert syndrome: Utility of mRNA and splicing studies. European Journal of Medical Genetics *63*, 104000. https://doi.org/10.1016/j.ejmg.2020.104000.
