# Supplementary material for: Exome sequencing early in outpatient evaluation in NCGENES 2: Changing the course of the diagnostic odyssey?
Source: HGG Adv. 2026 Apr 3;7(3):100605. doi: 10.1016/j.xhgg.2026.100605 (PMC13137186; doi:10.1016/j.xhgg.2026.100605)
Supplement: Document S2. Article plus supplemental information [file mmc3.pdf]

HGG Advances

ARTICLE

Exome sequencing early in outpatient evaluation in NCGENES 2: Changing the course of the diagnostic odyssey?

Tamara S. Roman,[1,2,5] Shannon M. Gray,[1,2,5] Tam P. Sneddon,[1,2] Ann Katherine M. Foreman,[1] Kristy Lee,[1] Cynthia M. Powell,[1,3] Karen E. Weck,[1,2] Jonathan S. Berg,[1] and Bradford C. Powell[1,4,6,*]

Summary

The impact of clinical exome and genome sequencing (ES/GS) depends on the clinical setting. In the sequencing arm of a multifactor randomized clinical trial to evaluate broadening access, we assessed the diagnostic and inconclusive findings of ES as a "first-tier" test in 101 pediatric participants in whom suspicion of a genetic condition by primary care providers had prompted initial outpatient consultation in a pediatric genetics or neurology clinic. This implementation focused on the early stages of the diagnostic odyssey, capturing a clinically less-selected population with lower pre-test probability than traditional specialist cohorts. Variants were prioritized using phenotype-driven gene lists. After returning the results to participants, the clinical teams performed familial variant testing or additional phenotyping at their discretion, based on potential diagnostic benefit. We then implemented a multidisciplinary case conference in which the laboratory and clinical teams assessed additional clinical information. Initially, 57% of participants had non-negative reports: 5% had one or more variant findings considered explanatory for the presenting phenotype, with 52% having results initially classified as inconclusive. After family testing and/or phenotypic characterization, a total of 9% were considered positive/diagnostic, 10% were reclassified from inconclusive to negative, and 38% remained inconclusive. While ES, as a first-tier genetic test, can expedite some diagnoses, these results demonstrate challenges in early implementation. In this population, initial testing can leave substantial residual uncertainty, shifting the diagnostic odyssey rather than concluding it and necessitating factors such as parental testing, ongoing phenotyping, and reassessment of variants over time to resolve inconclusive results.

Introduction

Genome-scale sequencing is becoming recognized as the standard of care for evaluating individuals in whom there is a high clinical suspicion of a variety of genetic conditions.[1–3] Abundant research has established the diagnostic yield of exome sequencing (ES) and genome sequencing (GS) in clinical contexts enriched for an underlying genetic diagnosis, including multiple congenital anomalies, intellectual disability, infants requiring neonatal intensive care, and pregnancies impacted by multiple fetal anomalies.[4–11] The American College of Medical Genetics and Genomics (ACMG) strongly recommends that ES/GS be considered as a first- or second-tier test for patients with congenital anomalies, developmental delay, or intellectual disability,[2] and others have also recommended ES/GS as first-tier testing for autism spectrum disorders[12] and in the evaluation of unexplained epilepsies.[13] It is likely that such testing may be increasingly ordered by non-clinical geneticist providers (e.g., pediatric neurologists or developmental pediatricians, as is the case for chromosomal microarray).[14]

With decreasing costs and increasing clinical availability of ES/GS, it is expected that earlier use may shorten the diagnostic odyssey. Expansion of access to ES/GS is hindered by limitations in access to genetics specialist services. This can place a substantial burden on patients and families seeking evaluation,[15] especially for patients facing other barriers to access care. A "sequencing-first" approach that expands ordering of genomic testing by primary care providers or other non-genetics specialists is one tool to address this.[16] However, the existing estimates of diagnostic yield of ES/GS, when ascertained in selected patients with high pre-test probability, may not generalize to patient groups that are less-stringently identified.[17–19] Notably, cohorts of patients much earlier in the diagnostic odyssey have been comparatively understudied. Diagnostic rates are higher in cohorts ascertained either based on domain-specific clinical expertise (i.e., after one or more evaluations by a clinician trained to recognize genetic conditions) or in populations whose disease type or severity is inherently enriched for individuals with monogenic conditions (e.g., patients in a neonatal intensive care unit or for whom inpatient genetics consultation is requested). For example, the Undiagnosed Disease

[1]Department of Genetics, University of North Carolina at Chapel Hill, Chapel Hill, NC 27599, USA; [2]Department of Pathology and Laboratory Medicine, University of North Carolina at Chapel Hill, Chapel Hill, NC 27599, USA; [3]Department of Pediatrics, University of North Carolina at Chapel Hill, Chapel Hill, NC 27599, USA
[4]Senior author
[5]These authors contributed equally
[6]Lead contact
*Correspondence: bpow@unc.edu
https://doi.org/10.1016/j.xhgg.2026.100605.
© 2026 The Author(s). Published by Elsevier Inc. on behalf of American Society of Human Genetics.
This is an open access article under the CC BY license (http://creativecommons.org/licenses/by/4.0/).

Network (UDN) has reported a diagnostic rate of ∼35%; however, this was after multidisciplinary evaluation and multimodality testing and in a selected cohort of 601 patients among the 1,519 who were referred for evaluation by that network, with preference given to acceptance of patients in whom diagnosis was most likely to be established.[20] Likewise, Mergnac et al. describe variability in diagnostic rate by indication, with an overall diagnostic rate of 55% among individuals referred to a reference center for inborn errors of metabolism, with much of this rate driven by osteogenesis imperfecta or specific metabolic disorders, including hyperlipidemia.[21] Wojcik et al. report an overall clinical diagnostic rate of 18.8% (including both monogenic and chromosomal conditions) over a 2-year follow-up period among children referred for diagnostic evaluation in a general genetic clinic.[15]

The proportion of patients undergoing genetic evaluation who truly have a genetic disease depends in part on the threshold of clinical suspicion required for referral. What is meant by “suspicion of a genetic condition” depends on the specific expertise of the clinician harboring the suspicion, and the threshold for referral to clinical genetics for evaluation is typically lower than the threshold for genetic testing; clinical geneticists have training to further refine the pre-test risk assessment and in many health care systems serve a “gatekeeping” role in deciding when genetic testing may be appropriate. In situations where reimbursement for genomic testing is low, this process may involve collecting additional data or awaiting the development of additional symptoms before proceeding with testing. Monogenic conditions may initially present in the outpatient setting with nonspecific signs that can have complex multifactorial etiologies. Because of this, performing genomic sequencing more broadly in patients who have these nonspecific findings may include a higher proportion of patients who do not have an underlying monogenic cause of their presentation.

If genome-scale sequencing information were available prior to or at initial genetic evaluation, either through reanalysis of sequence data obtained at birth or through testing ordered concurrently with a request for genetic consultation, how might this change the diagnostic odyssey? While this would be likely to shorten the time to diagnosis for many, it is possible that ES/GS testing at an earlier stage of evaluation could lead to additional difficulties in the interpretation of candidate variants, as the patient may not yet have developed other findings typically seen in a particular condition.[22,23] In such instances, ES/GS would not end the diagnostic odyssey, as additional evaluation might be required to provide clinical contextualization for inconclusive results.

The North Carolina Clinical Genomic Evaluation by Next-generation Exome Sequencing 2 (NCGENES 2) project[24] studied ES when implemented early in the evaluation of individuals whose primary care providers have referred them for an evaluation of a possible genetic condition, with goals of assessing educational interventions to prepare this cohort for genetic testing and to better understand factors that influence clinical usefulness in this setting. This study was part of the NIH-funded Clinical Sequencing Evidence-Generating Research (CSER2) consortium[25] that included collaborative working groups on many aspects of clinical sequencing implementation, including sequencing standards,[26] harmonization of measures,[27] data coordination,[28] variant classification,[29] community engagement,[30] and payer perspectives.[31]

The clinical project at the University of North Carolina (UNC) at Chapel Hill conducted a multi-site randomized controlled trial (ClinicalTrials.gov: NCT03548779) of ES for pediatric patients with suspected genetic disorders recruited at their initial consultation with a pediatric subspecialist (geneticist or neurologist). A factorial design was used for two independent interventions: (1) usual care vs. usual care with a pre-visit education intervention and (2) usual care vs. usual care with ES.[24] Participants in the second intervention arm underwent research ES at their initial consultation irrespective of the specialist clinician’s assessment of the chance of a genetic condition diagnosable by ES, thus allowing study of genomic testing in the absence of the traditional gatekeeping role of this evaluation. Primary outcomes examined the effect of these interventions on measures such as perceived patient centeredness and quality of life in each arm, as previously reported.[32] A secondary aim of NCGENES 2 was to characterize factors that affect clinical usefulness, uncertainty, and potential downstream costs if genome-scale sequencing were more widely implemented in this context. In this work, we focus on this secondary aim, describing the molecular findings of ES in 101 participants randomized to the ES arm of this CSER2 trial. These results include variant classification results, case-level interpretations, and instances where these classifications changed during the study period.

Subjects and methods

Patient recruitment

The NCGENES 2 study recruited participants from UNC in Chapel Hill, North Carolina; East Carolina State University (ECU) in Greenville, North Carolina; and Mission Hospital in Asheville, North Carolina. Details of the study criteria and design were previously described.[24] Parents of eligible pediatric participants (age <16 years, referred for a first outpatient visit at participating clinic for a suspected genetic condition, and having an eligible parent/guardian to participate in the clinic visit and study measures) were approached prior to the initial clinic visit. Enrollment occurred between October 2018 and August 2021, with data collection and analysis of clinical results through September 2023. The overall study involved activities and measures for pediatric participants, along with a parallel set of activities and measures for one designated parent or guardian per child. The parents who consented to study enrollment were randomized to (i) their participation in the child’s usual care or (ii) that participation in usual care plus a pre-visit educational preparation intervention.[32] Upon completion of their initial clinic visit, a separate consent

was performed for randomization to one of two arms with respect to ES: usual care or usual care plus offer of ES in the child participant at that clinic visit. Participants in the usual care arm could have had clinical ES performed if deemed clinically indicated by the treating team. Participants in the usual care plus offer of ES arm could decline that testing at any point. Those who received research ES were also provided with the option for an analysis of secondary findings. In addition to their standard-of-care clinical evaluation, the enrolling clinical geneticist provided descriptors of phenotypic features as Human Phenotype Ontology[33] (HPO) terms for later gene panel selection.

Ethics approval and consent to participate

This study was performed in accordance with the Declaration of Helsinki and was approved by the study's central institutional review board (IRB), the Biomedical IRB of the UNC, Chapel Hill, study no. 17-0816. Protocol modifications were reported to and approved by the UNC IRB. All participants (or parents/legal guardians for pediatric participants) provided informed consent before participating in this study, including consent for publication.

Exome Sequencing

DNA extraction, library preparation, sequencing, and primary bioinformatic analysis were performed as previously described.[24] Briefly, paired samples were obtained from each participant receiving ES, either as blood or saliva. One of these samples from each participant underwent extraction, library preparation using Agilent SureSelect XT (Human All Exon v.7), and sequencing on an Illumina HiSeq 4000 with a minimum average target depth of 50×. These sequencing data were then analyzed and annotated with reference data using an in-house-developed pipeline that included BWA-MEM[34] for mapping/alignment and FreeBayes[35] for calling genetic variants. The second sample was received by the CLIA-certified UNC McLendon Molecular Genetics Laboratory (MGL) for orthologous confirmation of reportable findings using Sanger sequencing.

Variant analysis

Variant analysis was performed on exome data using specific gene lists from Genomics England PanelApp,[36] which were applied individually to each study participant. These gene lists were selected by a study clinical or laboratory geneticist after review of the clinician-provided phenotypic feature terms and the documented reason for initial evaluation. To circumvent potential limitations of the *a priori* gene list approach, we also manually reviewed high-priority variants, which included variants predicted to be truncating or to affect splicing. Because of the relative nonspecificity of clinical findings in many participants, we often applied combined lists that include multiple primary PanelApp lists (for instance, "neurodevelopmental disorders" in 69 participants, "paediatric disorders" in 17 participants, "ophthalmological disorders" in 8 participants, and "neuromuscular disorders" in 3 participants; Table S1). In some instances, we manually searched the literature for genes associated with disease when a specific condition was high on the clinician's differential diagnosis. Additionally, we communicated with the enrolling clinicians to identify potential additional gene lists to analyze.

For participants who consented to the analysis and reporting of secondary findings, we also analyzed variants in the ACMG secondary findings gene list as current at the time of analysis. Variants identified by ES were classified into prioritization categories (high-priority variants included priority 1: reported as pathogenic [P] or likely pathogenic [LP] in ClinVar with at least a 2-star evidence rating; priority 2: minor-allele frequency [MAF] $\leq$ 0.01 and predicted truncating; and priority 3: MAF $\leq$ 0.01 and missense or other non-frameshift). After variant classification (using ACMG/AMP guidelines,[37] with customized guidance from ClinGen Expert Panel recommendations when available), exome results were discussed in weekly molecular sign-out meetings comprising genetic counselors, laboratory and clinical geneticists, researchers, and study leaders. The result of this discussion was the selection of variants based on a clear or possible relationship to the clinical indication or a secondary finding. The MGL performed variant confirmation by Sanger dideoxy sequencing and issued clinical reports for the confirmed variants, including releasing them to the Epic electronic health record for UNC patients.

Testing of parental samples to establish phase or *de novo* status was performed at the discretion of the enrolling clinician, based on their clinical impression of the likelihood that familial testing might clarify variant or case-level interpretation and when parents provided consent for this additional testing. Familial targeted Sanger sequencing was performed with the same primers used for variant confirmation in the probands.

Case-level interpretations and discussion

In addition to per-variant pathogenicity classifications, case-level interpretations (i.e., interpretations of genomic findings with respect to each participant's indication for referral) were made by incorporating information about the variants, the gene-disease association, and whether the gene-disease association was consistent with the phenotype of the study participant. These categories were a harmonized measure across the CSER consortium. Cases were interpreted as definitive positive when the implicated variants were classified as P, with documented zygosity, phase, or *de novo* status as expected for the described condition and a phenotype and inheritance pattern consistent with the associated condition. An interpretation of probable positive was assigned when similar criteria were met but at least one LP variant was implicated. Inconclusive cases involved findings that were deemed by the study's molecular analysis team to be reportable as a potential cause for further clinical consideration but with one or more contributions to case-level uncertainty. Individual categories of case-level uncertainty for these cases were recorded and could include unknown phase, variant uncertainty, insufficient zygosity, implication of phenotype expansion/contraction (phenotype mismatch), or lack of certainty of the gene-disease relationship (novel gene). To represent one case with discordance between the final laboratory classification and the clinician's contextualization, we added the category possible positive to represent a case that was inconclusive due to variant uncertainty but the clinician considered the result to be a likely explanation. This case was reported as inconclusive for consortium-level analyses. Cases without reportable results were categorized as negative. Initial case-level classifications were assigned by a study co-principal investigator (co-PI [B.C.P.]), informed by weekly molecular sign-out meeting discussions and the report that was issued by the clinical laboratory.

Periodic case conferences were held, allowing personnel of the molecular sign-out meetings to liaise with the enrolling clinicians (medical geneticists, neurologists, genetic counselors, and

advanced practice registered nurses). Case conferences were held separately with providers at the two institutions, which accounted for the majority of enrollments. All cases with one or more clinically confirmed and reported variants were reviewed with the enrolling clinicians from these institutions. Case conferences were not completed at the third site due to staff turnover and corresponding low participant recruitment at that site. Cases were typically discussed several months after results were issued to allow time for follow-up appointments and correlative studies as needed. Clinicians were informed of the cases to be discussed ahead of conferences. Discussion of each case began with a presentation from a variant analyst summarizing the variant analysis, the reported findings, and the initial case-level classification at the time of return of results. The results of family studies performed as part of this study were also reviewed. The clinicians then provided additional clinical context, including subsequent phenotype information and results of clinical genetic tests or other studies. When helpful, and with appropriate permission from families, clinical photographs were displayed. Each case concluded with a discussion that led to either endorsement or revision of the initial case-level result to arrive at a final consensus case-level interpretation. All conference attendees were welcome to participate in discussions, and we recorded both the consensus interpretation and the interpretation from the participant's physician, which were concordant in most cases.

Trial registration

This manuscript reports secondary outcomes within an arm of ClinicalTrials.gov: NCT03548779, registered at ClinicalTrials.gov on June 7, 2018.

Results

The NCGENES 2 study recruited a total of 403 pediatric participants. 250 of these participants completed the initial clinic visit and were eligible for randomization to usual care vs. usual care plus an offer of ES at that visit. Within the latter arm, the parents of 101 pediatric patients consented to ES for their children through this study. Subsequent parental Sanger sequencing was performed for 40 total individual parents to inform interpretation of proband sequence results. Among the 101 pediatric participants who were randomized to receive ES at their initial evaluation, 109 distinct variants were reported: 17 P, 20 LP, and 72 variants of uncertain significance (VUSs) (see Tables 1 and S1).

At the time of initial return of results, two participants (2%) had a definitive positive case-level interpretation (*PTEN* and *GJB2/GJB6*), three participants (3%) had a probable positive interpretation (*COL1A2*, *CSNK2B*, and *BRPF1*), 52 (51%) were inconclusive, and 44 (44%) participants had an initial negative classification. In addition, secondary findings[38] (*BRCA2*, *G6PD*, and *HFE*) were returned for 6 (5.9%) participants.

Case-level interpretations were recorded for the laboratory team at initial return of results and for the laboratory and clinician after discussion and review of additional information (Figure 1A). In a subset of case-level results initially classified by the laboratory as inconclusive, additional clinical information led to reclassification as negative (10 participants) or positive (5 participants) (Figure 1A). For individuals where additional clinical information could reclassify results clinically (Figure 1B), this included parental testing (6 participants), additional clinical evaluation (7 participants), or both (1 participant). The interval from initial report to reclassification based on additional clinical evaluation ranged from 5 to 22 months.

Diagnostic yield was measured by the proportion of cases with a final clinical interpretation of definitive, probable, or possible positive. Yield differed by the clinician's pre-test impression that ES would reveal a genetic cause for the participant's presentation, as rated by the clinical impression following the initial visit but before ES analysis (Table S1). Providers were asked to rate the likelihood of identifying a genetic cause at the initial visit on a 7-point Likert scale, scored from 0 = "extremely unlikely" to 6 = "extremely likely." The diagnostic yield was 6/12 (50%) among cases where the clinician rated this as extremely likely or "very likely" at the initial visit (rating 5 or 6), 4/76 (5.3%) where this was rated with an intermediate classification (rating 2, 3, or 4), 0/7 (0%) where this was rated as extremely unlikely or "very unlikely" (rating 0 or 1), and 0/6 (0%) when no response was given by the clinician.

The combination of factors contributing to case-level uncertainty in participants with inconclusive results is depicted in Figure 2. These factors were provided by phenotype category, with many participants' cases being evaluated in multiple categories and with respect to multiple variants. The most common factor implicated either by itself or in combination with other factors was variant uncertainty. Among 52 phenotype categories for initially inconclusive cases, 46 (88.5%) had variant uncertainty as at least one of the reasons why the case was inconclusive. The presence of variant uncertainty remained in 34/63 (54.0%) phenotypic categories in cases inconclusive at analysis completion. Only one case, which was classified as inconclusive due to an unknown phase, was able to be resolved by familial testing. Informative family members were not available for sequencing in the other 5 cases.

At the conclusion of this study, we identified 2 participants with a definitive positive result and 7 participants with a probable positive result, for an overall clinical diagnostic yield of 9 (9%) individuals with a definitive or probable positive result out of 101 total study participants. An additional 38 (38%) participants had results that remained clinically inconclusive at completion of the study.

In addition to the overall summary of reported results in Table S1, we provide details in the supplemental note for selected individuals whose case presentations highlight the issues we identified through qualitative consideration of the impact of sequencing early in the evaluation of suspected genetic conditions.

Table 1. Initial case-level classification of genetic testing results by sex, age, access-to-care categories, and broad indication categories

	Initial case-level classification (no. of participants)			
	Definitive positive	Probable positive	Inconclusive	Negative
**Sex**				
Female	0	1	17	19
Male	2	2	35	25
**Age at enrollment (years)**				
0–1	2	0	12	10
2–4	0	0	15	12
5–7	0	0	9	4
8–12	0	2	13	12
13–18	0	1	3	6
**Access to care**				
Served	0	1	11	15
Underserved	2	2	41	29
**Indication category**				
Central nervous system	0	0	0	2
Cancer	0	0	0	1
Dysmorphology	0	0	1	4
Metabolic	0	0	0	1
Neurodevelopmental	0	0	10	5
Neuromuscular	0	0	1	0
Skeletal dysplasia	0	1	1	2
Other	1	0	3	3
Multiple categories	1	2	36	26

Sex as listed here is as assigned to the participant at birth and consistent with chromosomal sex in all participants. Access-to-care categories are as defined in our protocol manuscript. The categories of indication for testing were harmonized measures and selected by the enrolling clinician at the initial evaluation. Multiple indications for testing were selectable. The total number of affected individuals was 101, with classifications based on all variants reported in those individuals. The number of variants reported in each individual ranged from 0 to 5.

Discussion

The NCGENES 2 study evaluated the effects of proband-only ES as an early testing modality in the evaluation of children referred for outpatient consultation due to a possible genetic condition. This study examined the diagnostic yield of ES in the pediatric setting and whether ES could shorten the overall diagnostic odyssey and/or lead to earlier application of medical management. This manuscript focuses on the diagnostic yield of genome-scale sequencing in the early evaluation of suspected genetic conditions.

The pre-test probability of a positive result is low at the initial outpatient visit but can be stratified through clinical expertise

The diagnostic yield found in our study was lower than has been reported in other studies, including others within the CSER2 consortium (SouthSeq GS: 30%[40]; NYCKidSeq GS: 16.5%[41]; and P3EGS pediatric cohort: 26.7%).[11] Acknowledging that factors in test methodology (ES vs. GS and proband vs. trio sequencing, as discussed below) could account for some differences, the degree of clinical selection for the underlying probability of a monogenic condition among the patients being sequenced also differed by study. For these other CSER2 studies, inclusion criteria involved either a pattern of congenital anomalies consistent with a syndromic cause or a major medical condition with no obvious etiology (SouthSeq), used phenotypic checklists typically filled out by study genetic counselors (NYCKidSeq), or enrolled based on having symptoms deemed suggestive of a genetic etiology by an enrolling genetic subspecialist (P3EGS). NCGENES 2, considering the implications of broader testing based on suspicion raised by a primary care provider, did not have the same level of clinical "pre-screening" prior to ES. After initial evaluation, the enrolling clinicians classified ES as "likely" to reveal a genetic cause for the clinical presentation in only 12 (12%)

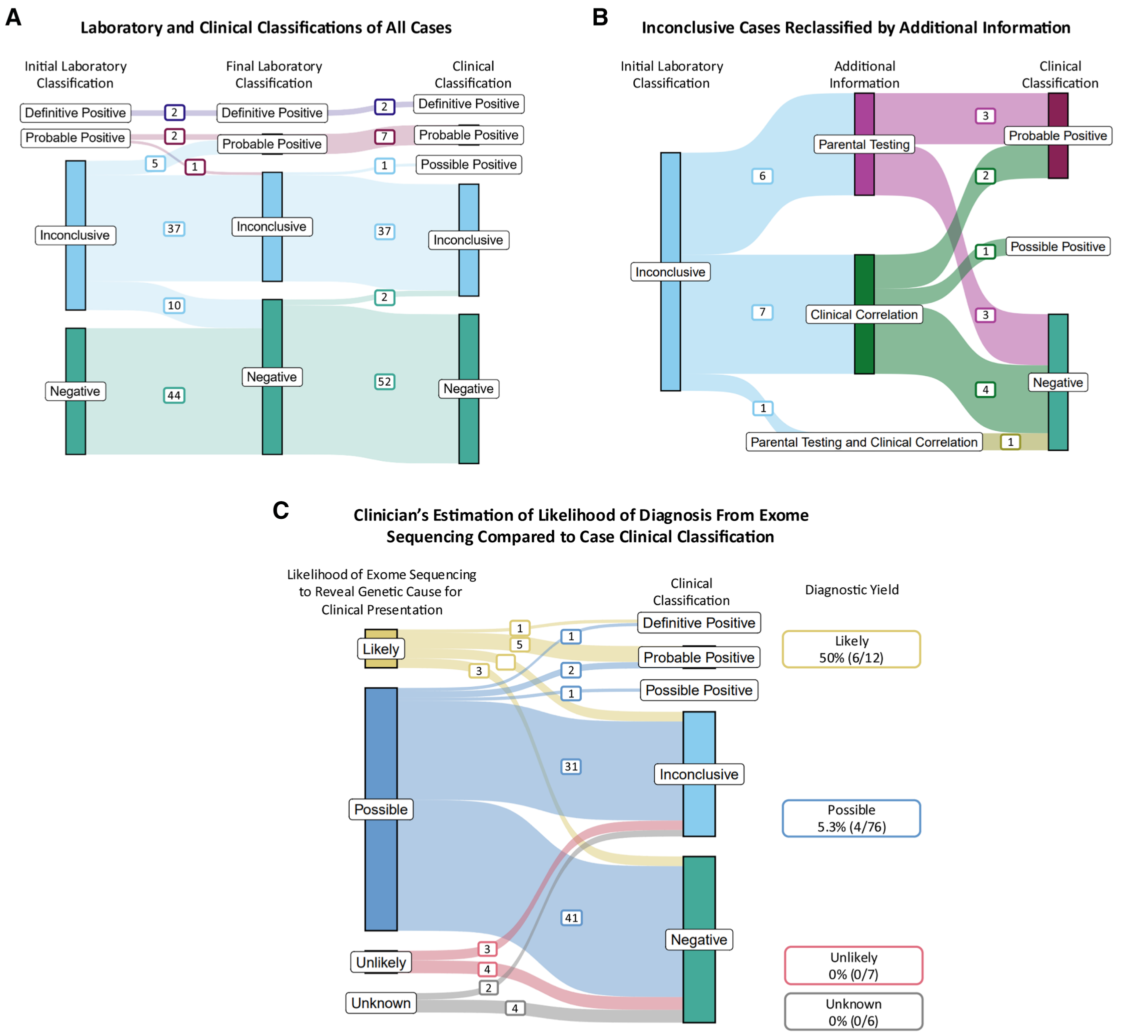

Figure 1. Visualization of individuals' case results over time as reclassified with additional clinical information or by clinician's post-evaluation assessment of likelihood of an identifiable genetic etiology
(A) Visualization of 101 case classifications at the initial laboratory level, final laboratory level, and clinical level.
(B) Visualization of cases initially classified as inconclusive by the laboratory and reclassified after the addition of parental testing information or clinical correlation. Note that cases classified as inconclusive both before and after parental testing and/or clinical correlation are omitted from this diagram.
(C) Comparison of enrolling clinicians' estimation of the likelihood of exome sequencing to reveal a genetic cause for the patient's clinical presentation as likely, possible, or unlikely. The diagnostic yield (percentage of definitive, probable, or possible positive results) is calculated within each of these categories.

participants (Figure 1C), and the rate of positive results was much higher among this group (6/12; 50%) than in the overall cohort. It is important that the clinician's impression of probability on a Likert scale should not be judged by an expectation of 100% accuracy, rather noting that clinician's impressions do provide valuable evidence to stratify likelihood and that a study with larger sample size might be able to demonstrate situations in which ES would not be warranted when the clinician's pre-test impression of a genetic condition is very low. Relevant to the consideration of genetics specialists' roles as gatekeepers to genomic sequencing, none of the participants for whom clinicians rated the likelihood of ES revealing a genetic cause as "unlikely" had results considered diagnostic, but 3 of these 7 participants (43%) had inconclusive results returned. The degree to which testing stratified by pre-test risk might be appropriate from the standpoint of utilization would depend on factors such as the cost of sequencing and potential downstream costs (e.g., need for additional phenotypic characterization to assess VUSs). Our study was not powered to perform a subgroup analysis of which indications for referral might lead to higher diagnostic yield in a sequencing-first approach, nor to which specific phenotypic characteristics guided clinicians' stratification of likelihood of ES revealing a genetic cause, but future studies of these questions could help inform the design of patterns of care for patients in whom primary care providers consider a genetic condition in the differential diagnosis.

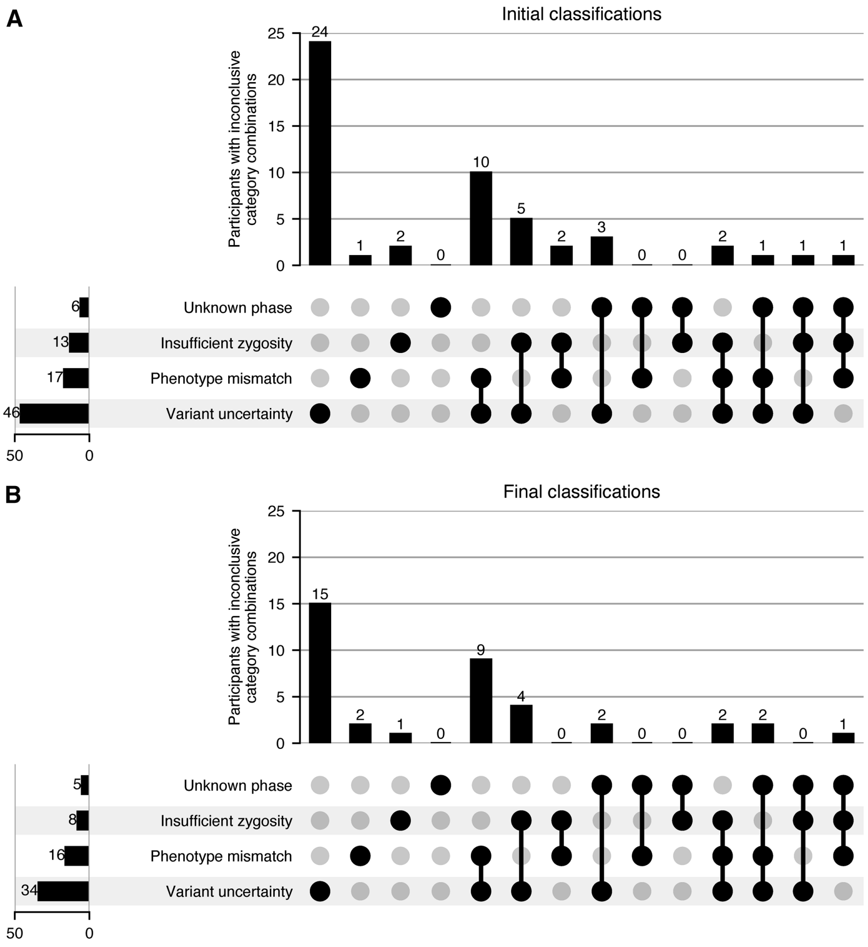

Figure 2. Factors contributing to inconclusive case results

For cases reported as inconclusive, the multidisciplinary sign-out committee recorded the reasons that variants with potential clinical relevance were not considered sufficient to provide a probable or definitive explanation of the indication for testing at the time of initial reporting (A) and the final laboratory interpretation after additional phenotyping and/or family testing and discussion with the enrolling clinician (B). The number of participants with each combination of factors is visualized using an UpSet[39] plot. The combination of factors shown here is aggregated across all reports for that participant and could involve inconclusive results for multiple variants and genes. Additional case-level details are provided in the supplemental note.

Benefits and limitations of using gene lists and ES to narrow down candidate variants

Using curated gene lists to filter variants of interest based on gene-disease associations and the participant's reported phenotype can help narrow down the list of variants for analysis and classification. Curated gene lists have implications for the efficiency and feasibility of genetic testing, but a gene list approach may limit sensitivity if variants of interest are in genes not present in the gene list. We used several techniques to mitigate this risk, as described in the subjects and methods, including expanding gene lists at the clinician's request and manual literature review when strongly suspected by the enrolling clinician.

We did not initially identify variants of interest in 4 participants because the variants were in genes not included on the PanelApp gene lists, as noted in the results section. A common scenario was a gene implicated in a newer or less well-understood monogenic disease relationship that was not included in the analysis. For example, the *TELO2* gene was not included in the PanelApp gene list at the time of analysis, and the reported variants in 15762386CSER were identified only through manual review after removing gene list filters. These variants would have been included as part of the filtered analysis using current versions of PanelApp gene lists. Likewise, in the absence of a Joubert-specific gene list in PanelApp, variants from 15846862CSER were analyzed using broader gene lists that nonetheless did not include *B9D1*, whereas a single heterozygous LP variant was identified through analysis of a literature-derived custom gene list. As an example of incomplete phenotypic information limiting search space, 15346997CSER had an additional reportable variant after the molecular analysis group was provided phenotypic information that was not part of the initial requisition, prompting evaluation of variants in additional genes. Similarly, we did not identify or report variants in *MC4R* in 15420762CSER that were subsequently reported from routine clinical testing because the participant had been enrolled with the primary phenotypic category of neurodevelopmental disorders, and our analysis did not include evaluation of genes with a more isolated association with early-onset obesity.

The selection of ES over GS in this study was based on lower sequencing cost (allowing more enrollment) and greater clinical availability. The literature indicates an incremental increase in the diagnostic yield of GS, with better identification of non-coding pathogenic variants and more accurate analysis of copy-number variants (CNVs) and other structural variation. However, insurance coverage for GS remains inconsistent relative to ES, attributed to the difference in costs and perceived insufficiency of evidence for the magnitude of differential clinical sensitivity by payors.[31,42]

Initial testing may be inconclusive for multiple reasons

While the ACMG and AMP provide guidelines[37] for classifying individual variant pathogenicity, they do not have

similar guidelines for the threshold of which results should be considered "reportable." Laboratory geneticists and molecular pathologists use a combination of variant classifications and available clinical information to make this determination, recognizing that a variant must be reported for the clinician to consider its presence in diagnostic thinking yet also acknowledging the potential for over-reporting to lead to unnecessary additional evaluation or other harms. By subdividing results deemed reportable but non-diagnostic, we quantify the different factors contributing to case-level uncertainty (Figure 2), which has implications for the likelihood of resolution. As anticipated, variant uncertainty was the largest contributor to inconclusive results, either alone or in combination with other factors. However, in this population where the "classic presentation" of a condition may not yet be evident because of the timing of testing, phenotype mismatch was the next most common contributor to inconclusive results.

Parental sequencing, when possible, is valuable for determining phase or *de novo* status

We performed ES on singleton study participants rather than on trios and conducted targeted follow-up variant sequencing for 40 parental samples. While recognizing the analytic benefits of proband/parent trio sequencing, this study design was based on the practicalities of sequencing in an outpatient setting, where lack of access to parental samples and differences in sequencing costs and reimbursement can hinder simultaneous sequencing of trios. This singleton sequencing approach impacted our ability to determine the phase and *de novo* status of variants during initial analysis. One challenge we encountered in this study, which also frequently occurs in clinical practice, is that parental samples may not be available for sequencing. This availability may depend on factors such as adoption, if a parent was deceased, or whether both parents consented to sequencing.

We identified multiple variants in the same genes for 8 participants (see Table S1). 15762386CSER, as described above, illustrates reclassification of variant-level and case-level results based on phase information. Parental sequencing could not be performed for 5 participants with one or more variants in *BSND*, *INTS1*, *KIAA1109*, *FAT4*, and *POLG*, which resulted in inconclusive case-level classifications due to phase uncertainty and/or insufficient zygosity.

While parental sequencing has value in establishing phase or *de novo* status, using inheritance information for variant classification is limited for conditions associated with reduced penetrance and/or variable expressivity, where unaffected or only mildly affected individuals could exist. This limitation is demonstrated with the *IFIH1* variant in 15762386CSER and the *GATA3* variant in 15197420CSER, in which classifications remained VUSs despite the determination that these variants were inherited from a parent. We did not perform follow-up parental sequencing for all reported VUSs; parental testing was deferred in cases where the enrolling clinician did not consider the VUS a reasonable explanation or potentially resolvable based on familial testing.

Phenotype characterization impacts the evaluation of variant and case-level results

The correlation of a molecular finding and the expected gene-phenotype association with the presenting symptoms of a patient contributes to the classification of variant pathogenicity.[43,44] Given that the clinical manifestations of many monogenic conditions evolve over time, patients undergoing genetic evaluation at an initial specialist visit may have phenotypic presentations that are less severe, less specific, and/or less complete than those presenting, for instance, with multiple congenital anomalies as neonates. Initiating genomic analysis at early time points may result in a shorter diagnostic odyssey for some and, for these individuals, reduce the need for diagnostic procedures performed over many years or "watchful waiting" until characteristic phenotypic features develop to guide specific testing. However, given the frequency of VUSs in genetic results, this approach may lead to results that are particularly difficult to contextualize in conditions associated with age-related penetrance, and younger patients may express limited phenotypic features.

Four participants were found to have variants in genes with only a partial association with the participant's phenotype (phenotype mismatch) or only considered a partial explanation by the clinician (additional clinical information for each is in the supplemental note). This represents an ongoing challenge in the interpretation of genome-scale sequencing, particularly when performed at earlier stages of evaluation, where the phenotype is less clear. Clinical correlation with the expected phenotypes of known diseases relies on both expression and observation of phenotypes in the patient undergoing testing, as well as documentation of the full spectrum of phenotypes for conditions from the available literature.

(1) In the case conference discussing 15254564CSER, an 8-year-old male with autism spectrum disorder, seizures, and spastic tetraplegic cerebral palsy, the enrolling clinician thought the *ATL1* LP variant may partially explain the patient's phenotype, given that *ATL1* pathogenic variants are associated with autosomal-dominant forms of spastic paraplegia and sensory neuropathy (MIM: 182600). The revised final case classification to probable positive was informed by the clinician's impression, recognizing that autism spectrum disorder has not been described in association with *ATL1*-related conditions and may represent a second condition in this participant.

(2) For 15316850CSER, a 15-year-old female with personal and family history of unexplained osteoporosis, the enrolling clinician described the patient's phenotype as not consistent with classic osteogenesis imperfecta, but the *COL1A2* LP variant could

explain some of her phenotype, noting that substitutions of glycine to small side-chain residues in the triple helical domain have been reported to result in substantial variability of expression.

(3) In 15504779CSER, a 3-year-old male with macrocephaly, autism spectrum disorder, and feeding difficulties, the precision of phenotype information available was not considered to have enough relationship to the typical presentation of the *PTEN* hamartoma tumor syndrome (MIM: 158350) to be able to establish this as the molecular etiology in the absence of prior reports of this variant.

(4) In 15640652CSER, a 14-year-old female referred for evaluation of osteogenesis imperfecta who was also noted to have maxillary lateral incisor microdontia, the clinician considered the *WNT10A* variant a partial explanation for this phenotype, and although no additional reportable variants were identified by ES, the possibility of dual diagnoses remains.[45,46] With microdontia being of less clinical prominence to the clinician and family, we classified the case result as inconclusive.

Additional information regarding variant classification and gene-disease associations over time contributes to molecular diagnoses

As sequencing studies increase in research and clinical testing, more information has been deposited into public databases, such as ClinVar,[47] and additional individuals and variants are identified in the context of genetic disease. The potential for obtaining additional molecular and clinical information underscores the value of performing periodic reassessments of variant classification and gene-disease associations in the context of specific phenotypes to resolve VUS results.[48] The final classifications of *CSNK2B* c.303C>G (GenBank: NM_001320.7) (p.Tyr101Ter [GenBank: NP_001311.3]) as LP for Poirier-Bienvenu neurodevelopmental syndrome (MIM: 618732) in 15151931CSER and of the *TELO2* c.392G>A (GenBank: NM_016111.4) (p.Gly131Asp [GenBank: NP_057195.2]) variant from a VUS to LP for You-Hoover-Fong syndrome (MIM: 616954) in 15762386CSER relied on additional literature reports of individuals with consistent phenotypes related to P variants in that gene (in the case of *CSNK2B*) or of the specific variant observed in the study participant (for *TELO2*). The importance of reassessing variant classification has been well established in clinical genetics. The use of case-level interpretation at different time points in this study, and more broadly in the CSER2 consortium, provides a framework to characterize how variant reclassification affects the case-level interpretability of results for patients.

Conclusions

"Early" genome-scale sequencing in the outpatient evaluation of children with suspected genetic conditions has the potential to shorten the diagnostic odyssey but in other instances may "change the course" of the odyssey by suggesting potential diagnoses that require additional evaluation. At the individual level, the effectiveness of genome-scale sequencing depends on whether the underlying etiology of the patient's condition is monogenic or multifactorial, and diagnostic yield is lower when sequencing is performed at this stage compared to testing in more highly selected patient populations. When patients with symptoms potentially suggestive of a monogenic disease undergo genome-scale sequencing, a "negative" result might be due to the lack of a monogenic etiology (and therefore a "true negative") or might simply be due to an underlying monogenic etiology remaining undiscovered (a "false negative"). When the population being sequenced is less enriched for patients with a high prior probability of a monogenic disease (as was the case in this study), the apparent diagnostic yield will be low, and a negative result should have high negative predictive value. Clinicians need to remain alert to current and future clinical and phenotypic features that would increase or decrease the patient's chance of having a monogenic disease (and therefore affect the clinical interpretation of a negative result).

There are other trade-offs to consider when employing genome-scale sequencing independent of a genetics specialist evaluation in patients with relatively nonspecific clinical features, such as developmental delay or neurobehavioral abnormalities. If the symptoms are truly due to an underlying monogenic disease, several factors can affect the diagnostic yield: (1) whether the genotype-phenotype relationship is well established at the time of molecular analysis, (2) whether sufficient phenotypic information is available to inform the evaluation of different loci and variants, and (3) whether samples are available for other family members to help with variant classification. The ability to use phenotypic information to limit the genomic search space for potentially causative variants is a critical factor in effective molecular analysis. The absence of key phenotypic details, whether due to the nature of the condition at early time points or lack of reporting by the clinician, may decrease recognition of relevant variants, thereby reducing clinical sensitivity. Simultaneously, genome-scale sequencing in patients with nonspecific phenotypes (especially those with potentially non-monogenic etiology, such as mild developmental delay and/or autism spectrum features) could result in multiple VUSs being reported due to potential phenotypic overlap. This would reduce clinical specificity and may prompt the need for additional evaluations that could alter the trajectory of the diagnostic odyssey for many individuals. Adequate phenotypic information at the time of molecular analysis, as well as iterative phenotyping to interpret the clinical significance of genome-scale results, is crucial. The periodic case conferences that we held with clinicians during the NCGENES 2 study often

highlighted the value of clinical phenotyping before and after genomic analysis.

While genomic sequencing has undoubtedly been established as an important part of the diagnostic work-up for many patients with suspected monogenic disease, developing tools to capture longitudinal phenotypic information and to quantify the pre-test and post-test probability of a monogenic disease over time will be essential for the most effective use of this diagnostic technology in clinical care. An ES/GS-first approach can improve accessibility to this technology for patients with identifiable genetic conditions in a health care system with limited access to genetics experts. This must be weighed against the lower diagnostic yield and increased burden of inconclusive results in scenarios where non-genetics experts are determining the likelihood that an individual has a genetic condition.

Data and code availability

Variant pathogenicity classifications from this project have been submitted to ClinVar with the submitter name "UNC Molecular Genetics Laboratory, University of North Carolina at Chapel Hill" and the study name "CSER_NCGENES_2." For subjects who consented to deidentified genomic data sharing, datasets are available in the access-restricted secure Analysis Visualization and Informatics Lab-space (AnVIL; #workspaces/anvil-datastorage/AnVIL_CSER_NCGENES2_GRU) and the Database of Genotypes and Phenotypes. The accession number for the data reported in this paper is dbGAP: phs002110.v1.p1.

Acknowledgments

NCGENES 2 was carried out as a collaborative study supported by the National Human Genome Research Institute, National Cancer Institute, and National Institute of Minority Health and Health Disparities of the National Institutes of Health for RFA-HG-12-009 under award number U01HG006487. The authors thank the CSER consortium and staff, advisory committees, and the clinicians and families participating in the NCGENES 2 study for their significant contributions. The authors would also like to acknowledge important contributions to this study made by the UNC High-Throughput Sequencing Facility (HTSF), the Renaissance Computing Institute (RENCI), the Mission Fullerton Genetics Lab, the Carolina Data Warehouse for Health, and the North Carolina Translational and Clinical Sciences (NCTRACS) Institute; Mai Xiong for Sanger sequencing confirmation of variants; and our study clinicians for case conference and discussion. We acknowledge Stefan Rentas, Dona Kanavy, and Tanner Coleman for contributions to variant analysis.

Author contributions

T.S.R., S.M.G., A.K.M.F., T.P.S., and B.C.P. wrote the manuscript. A.K.M.F., K.E.W., J.S.B., and B.C.P. made substantial contributions to the conception and design. T.S.R., S.M.G., A.K.M.F., T.P.S., C.M.P., K.E.W., J.S.B., and B.C.P. made substantial contributions to the acquisition, analysis, and interpretation of data. T.S.R., S.M.G., A.K.M.F., T.P.S., C.M.P., K.E.W., J.S.B., and B.C.P. were involved in drafting the manuscript or revising it critically for important intellectual content. All authors read and approved the final manuscript.

Declaration of interests

The authors declare no competing interests.

Supplemental information

Supplemental information can be found online at https://doi.org/10.1016/j.xhgg.2026.100605.

Web resources

ClinicalTrials.gov, https://clinicaltrials.gov
GenBank, https://www.ncbi.nlm.nih.gov/genbank/
Online Mendelian Inheritance in Man (OMIM), https://omim.org

Received: November 13, 2025
Accepted: April 1, 2026

References

1. Berg, J.S. (2014). Genome-Scale Sequencing in Clinical Care: Establishing Molecular Diagnoses and Measuring Value. JAMA *312*, 1865–1867. https://doi.org/10.1001/jama.2014.14665.
2. Manickam, K., McClain, M.R., Demmer, L.A., Biswas, S., Kearney, H.M., Malinowski, J., Massingham, L.J., Miller, D., Yu, T.W., Hisama, F.M.; and ACMG Board of Directors (2021). Exome and genome sequencing for pediatric patients with congenital anomalies or intellectual disability: an evidence-based clinical guideline of the American College of Medical Genetics and Genomics (ACMG). Genet. Med. *23*, 2029–2037. https://doi.org/10.1038/s41436-021-01242-6.
3. Freed, A.S., Clowes Candadai, S.V., Sikes, M.C., Thies, J., Byers, H.M., Dines, J.N., Ndugga-Kabuye, M.K., Smith, M.B., Fogus, K., Mefford, H.C., et al. (2020). The Impact of Rapid Exome Sequencing on Medical Management of Critically Ill Children. J. Pediatr. *226*, 202–212.e1. https://doi.org/10.1016/j.jpeds.2020.06.020.
4. Retterer, K., Juusola, J., Cho, M.T., Vitazka, P., Millan, F., Gibellini, F., Vertino-Bell, A., Smaoui, N., Neidich, J., Monaghan, K.G., et al. (2016). Clinical application of whole-exome sequencing across clinical indications. Genet. Med. *18*, 696–704. https://doi.org/10.1038/gim.2015.148.
5. Smith, H.S., Swint, J.M., Lalani, S.R., Yamal, J.-M., De Oliveira Otto, M.C., Castellanos, S., Taylor, A., Lee, B.H., and Russell, H.V. (2019). Clinical Application of Genome and Exome Sequencing as a Diagnostic Tool for Pediatric Patients: a Scoping Review of the Literature. Genet. Med. *21*, 3–16. https://doi.org/10.1038/s41436-018-0024-6.
6. Malinowski, J., Miller, D.T., Demmer, L., Gannon, J., Pereira, E.M., Schroeder, M.C., Scheuner, M.T., Tsai, A.C.-H., Hickey, S.E., Shen, J.; and ACMG Professional Practice and Guidelines Committee (2020). Systematic evidence-based review: outcomes from exome and genome sequencing for pediatric patients with congenital anomalies or intellectual disability. Genet. Med. *22*, 986–1004. https://doi.org/10.1038/s41436-020-0771-z.

7. Shickh, S., Mighton, C., Uleryk, E., Pechlivanoglou, P., and Bombard, Y. (2021). The clinical utility of exome and genome sequencing across clinical indications: a systematic review. Hum. Genet. *140*, 1403–1416. https://doi.org/10.1007/s00439-021-02331-x.
8. Stefanski, A., Calle-López, Y., Leu, C., Pérez-Palma, E., Pestana-Knight, E., and Lal, D. (2021). Clinical sequencing yield in epilepsy, autism spectrum disorder, and intellectual disability: A systematic review and meta-analysis. Epilepsia *62*, 143–151. https://doi.org/10.1111/epi.16755.
9. Mellis, R., Oprych, K., Scotchman, E., Hill, M., and Chitty, L.S. (2022). Diagnostic yield of exome sequencing for prenatal diagnosis of fetal structural anomalies: A systematic review and meta-analysis. Prenat. Diagn. *42*, 662–685. https://doi.org/10.1002/pd.6115.
10. Muriello, M. (2022). Exome and Whole Genome Sequencing in the Neonatal Intensive Care Unit. Clin. Perinatol. *49*, 167–179. https://doi.org/10.1016/j.clp.2021.11.018.
11. Slavotinek, A., Rego, S., Sahin-Hodoglugil, N., Kvale, M., Lianoglou, B., Yip, T., Hoban, H., Outram, S., Anguiano, B., Chen, F., et al. (2023). Diagnostic yield of pediatric and prenatal exome sequencing in a diverse population. npj Genom. Med. *8*, 10. https://doi.org/10.1038/s41525-023-00353-0.
12. Arteche-López, A., Gómez Rodríguez, M.J., Sánchez Calvin, M.T., Quesada-Espinosa, J.F., Lezana Rosales, J.M., Palma Milla, C., Gómez-Manjón, I., Hidalgo Mayoral, I., Pérez de la Fuente, R., Díaz de Bustamante, A., et al. (2021). Towards a Change in the Diagnostic Algorithm of Autism Spectrum Disorders: Evidence Supporting Whole Exome Sequencing as a First-Tier Test. Genes *12*, 560. https://doi.org/10.3390/genes12040560.
13. Smith, L., Malinowski, J., Ceulemans, S., Peck, K., Walton, N., Sheidley, B.R., and Lippa, N. (2023). Genetic testing and counseling for the unexplained epilepsies: An evidence-based practice guideline of the National Society of Genetic Counselors. J. Genet. Couns. *32*, 266–280. https://doi.org/10.1002/jgc4.1646.
14. Miller, D.T., Adam, M.P., Aradhya, S., Biesecker, L.G., Brothman, A.R., Carter, N.P., Church, D.M., Crolla, J.A., Eichler, E.E., Epstein, C.J., et al. (2010). Consensus Statement: Chromosomal Microarray Is a First-Tier Clinical Diagnostic Test for Individuals with Developmental Disabilities or Congenital Anomalies. Am. J. Hum. Genet. *86*, 749–764. https://doi.org/10.1016/j.ajhg.2010.04.006.
15. Wojcik, M.H., Bresnahan, M., del Rosario, M.C., Ojeda, M.M., Kritzer, A., and Fraiman, Y.S. (2023). Rare diseases, common barriers: disparities in pediatric clinical genetics outcomes. Pediatr. Res. *93*, 110–117. https://doi.org/10.1038/s41390-022-02240-3.
16. Curd, H., Gorrie, A., and Fennell, A.P. (2025). From tradition to transformation: evolving models of care in clinical genetics. Curr. Opin. Pediatr. *37*, 538–549. https://doi.org/10.1097/MOP.0000000000001502.
17. 100000 Genomes Project Pilot Investigators, Smedley, D., Smith, K.R., Martin, A., Thomas, E.A., McDonagh, E.M., Cipriani, V., Ellingford, J.M., Arno, G., Tucci, A., et al. (2021). 100,000 Genomes Pilot on Rare-Disease Diagnosis in Health Care — Preliminary Report. N. Engl. J. Med. *385*, 1868–1880. https://doi.org/10.1056/NEJMoa2035790.
18. Brockman, D.G., Austin-Tse, C.A., Pelletier, R.C., Harley, C., Patterson, C., Head, H., Leonard, C.E., O'Brien, K., Mahanta, L.M., Lebo, M.S., et al. (2021). Randomized prospective evaluation of genome sequencing versus standard-of-care as a first molecular diagnostic test. Genet. Med. *23*, 1689–1696. https://doi.org/10.1038/s41436-021-01193-y.
19. Evans, J.P., Powell, B.C., and Berg, J.S. (2017). Finding the Rare Pathogenic Variants in a Human Genome. JAMA *317*, 1904–1905. https://doi.org/10.1001/jama.2017.0432.
20. Splinter, K., Adams, D.R., Bacino, C.A., Bellen, H.J., Bernstein, J.A., Cheatle-Jarvela, A.M., Eng, C.M., Esteves, C., Gahl, W.A., Hamid, R., et al. (2018). Effect of Genetic Diagnosis on Patients with Previously Undiagnosed Disease. N. Engl. J. Med. *379*, 2131–2139. https://doi.org/10.1056/NEJMoa1714458.
21. Mergnac, J.-P., Wiedemann, A., Chery, C., Ravel, J.-M., Namour, F., Guéant, J.-L., Feillet, F., and Oussalah, A. (2022). Diagnostic yield of clinical exome sequencing as a first-tier genetic test for the diagnosis of genetic disorders in pediatric patients: results from a referral center study. Hum. Genet. *141*, 1269–1278. https://doi.org/10.1007/s00439-021-02358-0.
22. Klee, E.W., Cousin, M.A., Pinto E Vairo, F., Morales-Rosado, J.A., Macke, E.L., Jenkinson, W.G., Ferrer, A., Schultz-Rogers, L.E., Olson, R.J., Oliver, G.R., et al. (2021). Impact of integrated translational research on clinical exome sequencing. Genet. Med. *23*, 498–507. https://doi.org/10.1038/s41436-020-01005-9.
23. Elliott, A.M., Adam, S., Du Souich, C., Lehman, A., Nelson, T.N., Van Karnebeek, C., Alderman, E., Armstrong, L., Aubertin, G., Blood, K., et al. (2022). Genome-wide sequencing and the clinical diagnosis of genetic disease: The CAUSES study. HGG Adv. *3*, 100108. https://doi.org/10.1016/j.xhgg.2022.100108.
24. Staley, B.S., Milko, L.V., Waltz, M., Griesemer, I., Mollison, L., Grant, T.L., Farnan, L., Roche, M., Navas, A., Lightfoot, A., et al. (2021). Evaluating the clinical utility of early exome sequencing in diverse pediatric outpatient populations in the North Carolina Clinical Genomic Evaluation of Next-generation Exome Sequencing (NCGENES) 2 study: a randomized controlled trial. Trials *22*, 395. https://doi.org/10.1186/s13063-021-05341-2.
25. Amendola, L.M., Berg, J.S., Horowitz, C.R., Angelo, F., Bensen, J.T., Biesecker, B.B., Biesecker, L.G., Cooper, G.M., East, K., Filipski, K., et al. (2018). The Clinical Sequencing Evidence-Generating Research Consortium: Integrating Genomic Sequencing in Diverse and Medically Underserved Populations. Am. J. Hum. Genet. *103*, 319–327. https://doi.org/10.1016/j.ajhg.2018.08.007.
26. Sanghvi, R.V., Buhay, C.J., Powell, B.C., Tsai, E.A., Dorschner, M.O., Hong, C.S., Lebo, M.S., Sasson, A., Hanna, D.S., McGee, S., et al. (2018). Characterizing reduced coverage regions through comparison of exome and genome sequencing data across 10 centers. Genet. Med. *20*, 855–866. https://doi.org/10.1038/gim.2017.192.
27. Goddard, K.A.B., Angelo, F.A.N., Ackerman, S.L., Berg, J.S., Biesecker, B.B., Danila, M.I., East, K.M., Hindorff, L.A., Horowitz, C.R., Hunter, J.E., et al. (2020). Lessons learned about harmonizing survey measures for the CSER consortium.

J. Clin. Transl. Sci. *4*, 537–546. https://doi.org/10.1017/cts.2020.41.

28. Muenzen, K.D., Amendola, L.M., Kauffman, T.L., Mittendorf, K.F., Bensen, J.T., Chen, F., Green, R., Powell, B.C., Kvale, M., Angelo, F., et al. (2022). Lessons learned and recommendations for data coordination in collaborative research: The CSER consortium experience. HGG Adv. *3*, 100120. https://doi.org/10.1016/j.xhgg.2022.100120.
29. Amendola, L.M., Muenzen, K., Biesecker, L.G., Bowling, K.M., Cooper, G.M., Dorschner, M.O., Driscoll, C., Foreman, A.K.M., Golden-Grant, K., Greally, J.M., et al. (2020). Variant Classification Concordance using the ACMG-AMP Variant Interpretation Guidelines across Nine Genomic Implementation Research Studies. Am. J. Hum. Genet. *107*, 932–941. https://doi.org/10.1016/j.ajhg.2020.09.011.
30. Griesemer, I., Staley, B.S., Lightfoot, A.F., Bain, L., Byrd, D., Conway, C., Grant, T.L., Leach, B., Milko, L., Mollison, L., et al. (2020). Engaging community stakeholders in research on best practices for clinical genomic sequencing. Per. Med. *17*, 435–444. https://doi.org/10.2217/pme-2020-0074.
31. Phillips, K.A., Trosman, J.R., Douglas, M.P., Gelb, B.D., Ferket, B.S., Hindorff, L.A., Slavotinek, A.M., Berg, J.S., Russell, H.V., Devine, B., et al. (2022). US private payers' perspectives on insurance coverage for genome sequencing versus exome sequencing: A study by the Clinical Sequencing Evidence-Generating Research Consortium (CSER). Genet. Med. *24*, 238–244. https://doi.org/10.1016/j.gim.2021.08.009.
32. Waltz, M., Yan, H., Cadigan, R.J., Canter, C., Bain, L., Bensen, J.T., Conway, C., Haldeman-Englert, C., Farnan, L., M Foreman, A.K., et al. (2023). Question prompt lists and caregiver question asking in pediatric specialty appointments: A randomized controlled trial. Patient Educ. Couns. *109*, 107620. https://doi.org/10.1016/j.pec.2022.107620.
33. Köhler, S., Gargano, M., Matentzoglu, N., Carmody, L.C., Lewis-Smith, D., Vasilevsky, N.A., Danis, D., Balagura, G., Baynam, G., Brower, A.M., et al. (2021). The Human Phenotype Ontology in 2021. Nucleic Acids Res. *49*, D1207–D1217. https://doi.org/10.1093/nar/gkaa1043.
34. Li, H. (2013). Aligning sequence reads, clone sequences and assembly contigs with BWA-MEM. Preprint at arXiv. https://doi.org/10.48550/arXiv.1303.3997.
35. Garrison, E., and Marth, G. (2012). Haplotype-based variant detection from short-read sequencing. Preprint at arXiv. https://doi.org/10.48550/arXiv.1207.3907.
36. Martin, A.R., Williams, E., Foulger, R.E., Leigh, S., Daugherty, L.C., Niblock, O., Leong, I.U.S., Smith, K.R., Gerasimenko, O., Haraldsdottir, E., et al. (2019). PanelApp crowdsources expert knowledge to establish consensus diagnostic gene panels. Nat. Genet. *51*, 1560–1565. https://doi.org/10.1038/s41588-019-0528-2.
37. Richards, S., Aziz, N., Bale, S., Bick, D., Das, S., Gastier-Foster, J., Grody, W.W., Hegde, M., Lyon, E., Spector, E., et al. (2015). Standards and guidelines for the interpretation of sequence variants: a joint consensus recommendation of the American College of Medical Genetics and Genomics and the Association for Molecular Pathology. Genet. Med. *17*, 405–424. https://doi.org/10.1038/gim.2015.30.
38. Miller, D.T., Lee, K., Chung, W.K., Gordon, A.S., Herman, G.E., Klein, T.E., Stewart, D.R., Amendola, L.M., Adelman, K., Bale, S.J., et al. (2021). ACMG SF v3.0 list for reporting of secondary findings in clinical exome and genome sequencing: a policy statement of the American College of Medical Genetics and Genomics (ACMG). Genet. Med. *23*, 1381–1390. https://doi.org/10.1038/s41436-021-01172-3.
39. Lex, A., Gehlenborg, N., Strobelt, H., Vuillemot, R., and Pfister, H. (2014). UpSet: Visualization of Intersecting Sets. IEEE Trans. Vis. Comput. Graph. *20*, 1983–1992. https://doi.org/10.1109/TVCG.2014.2346248.
40. Bowling, K.M., Thompson, M.L., Finnila, C.R., Hiatt, S.M., Latner, D.R., Amaral, M.D., Lawlor, J.M.J., East, K.M., Cochran, M.E., Greve, V., et al. (2022). Genome sequencing as a first-line diagnostic test for hospitalized infants. Genet. Med. *24*, 851–861. https://doi.org/10.1016/j.gim.2021.11.020.
41. Abul-Husn, N.S., Marathe, P.N., Kelly, N.R., Bonini, K.E., Sebastin, M., Odgis, J.A., Abhyankar, A., Brown, K., Di Biase, M., Gallagher, K.M., et al. (2023). Molecular diagnostic yield of genome sequencing versus targeted gene panel testing in racially and ethnically diverse pediatric patients. Genet. Med. *25*, 100880. https://doi.org/10.1016/j.gim.2023.100880.
42. Zion, T.N., Berrios, C.D., Cohen, A.S.A., Bartik, L., Cross, L.A., Engleman, K.L., Fleming, E.A., Gadea, R.N., Hughes, S.S., Jenkins, J.L., et al. (2023). Insurance denials and diagnostic rates in a pediatric genomic research cohort. Genet. Med. *25*, 100020. https://doi.org/10.1016/j.gim.2023.100020.
43. Pinto E Vairo, F., Kemppainen, J.L., Vitek, C.R.R., Whalen, D.A., Kolbert, K.J., Sikkink, K.J., Kroc, S.A., Kruisselbrink, T., Shupe, G.F., Knudson, A.K., et al. (2023). Implementation of genomic medicine for rare disease in a tertiary healthcare system: Mayo Clinic Program for Rare and Undiagnosed Diseases (PRaUD). J. Transl. Med. *21*, 410. https://doi.org/10.1186/s12967-023-04183-7.
44. Ediae, G.U., Lemire, G., Chisholm, C., Hartley, T., Eaton, A., Osmond, M., Rojas, S.K., Huang, L., Gillespie, M., Care4Rare Canada Consortium, et al. (2023). The implementation of an enhanced clinical model to improve the diagnostic yield of exome sequencing for patients with a rare genetic disease: A Canadian experience. Am. J. Med. Genet. *191*, 338–347. https://doi.org/10.1002/ajmg.a.63022.
45. Posey, J.E., Harel, T., Liu, P., Rosenfeld, J.A., James, R.A., Coban Akdemir, Z.H., Walkiewicz, M., Bi, W., Xiao, R., Ding, Y., et al. (2017). Resolution of Disease Phenotypes Resulting from Multilocus Genomic Variation. N. Engl. J. Med. *376*, 21–31. https://doi.org/10.1056/NEJMoa1516767.
46. Smith, E.D., Blanco, K., Sajan, S.A., Hunter, J.M., Shinde, D.N., Wayburn, B., Rossi, M., Huang, J., Stevens, C.A., Muss, C., et al. (2019). A retrospective review of multiple findings in diagnostic exome sequencing: half are distinct and half are overlapping diagnoses. Genet. Med. *21*, 2199–2207. https://doi.org/10.1038/s41436-019-0477-2.
47. Landrum, M.J., Lee, J.M., Benson, M., Brown, G.R., Chao, C., Chitipiralla, S., Gu, B., Hart, J., Hoffman, D., Jang, W., et al. (2018). ClinVar: improving access to variant interpretations and supporting evidence. Nucleic Acids Res. *46*, D1062–D1067. https://doi.org/10.1093/nar/gkx1153.
48. Liu, P., Meng, L., Normand, E.A., Xia, F., Song, X., Ghazi, A., Rosenfeld, J., Magoulas, P.L., Braxton, A., Ward, P., et al. (2019). Reanalysis of Clinical Exome Sequencing Data. N. Engl. J. Med. *380*, 2478–2480. https://doi.org/10.1056/NEJMc1812033.

HGGA, Volume 7

Supplemental information

Exome sequencing early in outpatient evaluation in NCGENES 2: Changing the course of the diagnostic odyssey?

Tamara S. Roman, Shannon M. Gray, Tam P. Sneddon, Ann Katherine M. Foreman, Kristy Lee, Cynthia M. Powell, Karen E. Weck, Jonathan S. Berg, and Bradford C. Powell

Supplemental Note: Case Reports

15151931CSER: an 8-year-old male referred with hearing loss, mild intellectual disability and seizure disorders. A heterozygous *CSNK2B* NM_001320.7:c.303C>G NP_001311.3:p.(Tyr101Ter) variant was identified. At initial molecular analysis, pathogenic *CSNK2B* variation was not well characterized in the literature, limited to a few previous case reports.[1–3] The phenotypes of that series of patients included developmental delay, mild facial dysmorphism, and epilepsy. Since our initial analysis, pathogenic *CSNK2B* variants have since been reported in multiple studies of individuals with intellectual disability with or without epilepsy,[4,5] and *CSNK2B* is classified as definitively associated with autosomal dominant Poirier-Bienvenu neurodevelopmental syndrome [MIM: 618732] by ClinGen.[6]

15197420CSER: a 4-year-old male enrolled with a primary indication of unspecified hearing loss, also noted to have growth and developmental delays. ES revealed a variant in *GATA3*: NM_001002295.2:c.1256C>T NP_001002295.1:p.(Thr419Met), classified as a Variant of Uncertain Significance (VUS) for *GATA3*-associated hypoparathyroidism, sensorineural deafness, and renal dysplasia [MIM: 146255]. Despite a relatively high population allele frequency (0.003758 in the admixed American population in gnomAD v2.1.1), we considered the reported phenotypic variability and age-dependent manifestations of this condition and reported this variant as a VUS with possible contribution to the patient's phenotype. Parental testing was performed since *de novo* status would be sufficient for reclassification, but the variant was shown to be paternally inherited. The father had a reported history of speech delay but no documented hearing loss and normal renal ultrasound. Endocrine

evaluation of the father was recommended. Considering the possibility of variability of expression and the incomplete phenotypic information available for the father, we could not exclude the *GATA3* variant as causative, so the case-level result was inconclusive due to variant uncertainty.

15254564CSER: a 8-year-old male with autism spectrum disorder, seizures and spastic tetraplegic cerebral palsy. Five variants were reported after exome variant analysis: 3 VUS in *TECTA*, *TRIO* and *ZMYND11* and 2 Likely Pathogenic (LP) variants in *ATL1* and *DHPS*. *ATL1* has been associated with adult-onset hereditary sensory neuropathy type 1D [MIM: 613708] and Spastic paraplegia 3A [MIM: 182600], both as autosomal dominant conditions. With subsequent clinical evaluation, the patient was noted to have more pronounced facial asymmetry, developmental delay, and finger pads. The evaluating clinician considered the *ATL1* finding as probable positive with respect to the participant's spasticity but not explanatory of the patients' other phenotypes.

15316850CSER: a 15-year-old female with a personal and family history (in her sister) of idiopathic osteoporosis. She was found to have a heterozygous *COL1A2* NM_000089.4:c.964G>A NP_000080.2:p.(Gly322Ser) LP variant which was returned as a probable positive case-level result for *COL1A2*-related Osteogenesis Imperfecta. Testing of the participant's sister detected the same *COL1A2* variant. Phenotypically, these patients are most consistent with mild Osteogenesis Imperfecta.

15346997CSER: a 9-month-old referred with short stature, bilateral blurry vision, and encephalopathy. Variants within genes on PanelApp Neurodevelopmental Disorders and Ophthalmological Disorders lists included four classified as VUS in

different genes with possible relation to the patient's phenotype (Supplemental Table 1). Further discussion with the enrolling clinician and additional phenotype information in the study participant prompted a request for analysis of genes associated with Ehlers-Danlos syndromes. This re-analysis identified a VUS in *COL6A2,* in which pathogenic variants have been associated with varying degrees of myopathy and contractures (Bethlem myopathy 1B [MIM: 620725] and Ullrich congenital muscular dystrophy 1B [MIM:620727]). This variant, NM_001849.4:c.139G>A NP_001840.3:p.(Val47Met), was reported as possibly related to the phenotype in this inconclusive case.

15420762CSER: a 12-year-old female enrolled with thin upper lip vermilion, bulbous nose, upslanted palpebral fissure, specific learning disability, growth hormone deficiency, and obesity. Variants identified by exome sequencing were filtered using the PanelApp Neurodevelopment Disorders gene list. A separate obesity gene panel conducted as part of this participant's usual clinical care identified two *MC4R* variants associated with autosomal dominant childhood-onset obesity: NM_005912.3:c.105C>A NP_005903.2:p.(Tyr35Ter) and NM_005912.3:c.110A>T NP_005903.2:p.(Asp37Val). These variants were reportedly *in cis.* However, because *MC4R* is not included on the PanelApp Neurodevelopment Disorders gene list, these two variants were not reviewed as part of the exome molecular analysis process. Of note, *MC4R* is included on the PanelApp Severe Early-onset Obesity gene list. With respect to the participant's presentation, this result is classified inconclusive, since the *MC4R* findings may only explain the patient's obesity, while other aspects of her phenotype remain unexplained.

15504779CSER: a 3-year-old male with macrocephaly (> 97th percentile), autism spectrum disorder and feeding difficulties. Sequencing detected a heterozygous *PTEN*

NM_000314.8:c.752G>A NP_000305.3:p.(Gly251Asp) VUS. While this variant has been reported previously in the somatic context in a papillary tumor of the pineal region[7], to our knowledge it has not been reported in affected individuals with *PTEN* hamartoma tumor syndrome [MIM: 158350]. We ultimately classified this *PTEN* variant as a VUS due to insufficient phenotypic data available for this study participant, as well as the lack of evidence of a deleterious fitness score in an in vitro functional assay.[8] Thus, the case-level result was deemed inconclusive.

15600677CSER: a 4-year-old female enrolled with developmental delay, hypotonia, and dysmorphic facial features. Sequencing identified two heterozygous missense variants, both reported as VUS: *DAG1* NM_004393.6:c.2194C>A NP_004384.5:p.(Pro732Thr) and *SOX5* NM_006940.6:c.1673G>A NP_008871.3:p.(Arg558His). Case-level results were reported as inconclusive, but the enrolling clinician considered *SOX5*-related Lamb-Shaffer syndrome [MIM: 616803] to be a reasonable fit for the participant's presentation. The parents of the participant consented to targeted sequencing which established the *SOX5* variant to be *de novo*; this provided additional evidence to upgrade the *SOX5* variant to LP, ultimately changing the case-level classification from inconclusive to probable positive.

15640652CSER: a 14-year-old female referred because of osteogenesis imperfecta. She was noted at enrollment to have maxillary lateral incisor microdontia. We identified a heterozygous *WNT10A* NM_025216.3:c.321C>A NP_079492.2:p.(Cys107Ter) pathogenic variant, known to be associated with a spectrum of phenotypes, ranging from isolated oligodontia to complex forms of autosomal recessive ectodermal dysplasia. This finding is a likely explanation for the

patient's microdontia. However, since the primary indication for testing was a history of recurrent fractures of the long bones, a phenotype not associated with loss-of-function *WNT10A* variants, the case-level interpretation was considered inconclusive with respect to the participant's reason for presentation.

15762386CSER: a 2-year-old male referred with microcephaly, low weight for age, and developmental delay. Three variants of potential relevance (Supplemental Table 1) were identified through filtering for genes in PanelApp Severe Microcephaly and Neurodevelopmental Disorders gene lists, with none of these results considered sufficient to definitively explain the phenotype. One of these findings was a heterozygous variant in *IFIH1*: NM_022168.4:c.2759T>C NP_071451.2:p.(Ile920Thr) classified as a VUS for Aicardi-Goutieres syndrome [MIM: 615846]. Additional manual review of variants outside of the designated gene lists identified two heterozygous missense variants in *TELO2*: NM_016111.4:c.392G>A NP_057195.2:p.(Gly131Asp), classified as a VUS, and NM_016111.4:c.1100G>T NP_057195.2:p.(Cys367Phe), classified as LP for You-Hoover-Fong syndrome [MIM: 616954] (*TELO2* has since been added to multiple PanelApp lists, including the Neurodevelopmental Disorders list). The *IFIH1* variant was maternally inherited; however, given literature evidence of incomplete penetrance in Aicardi-Goutieres syndrome, the presence of this variant in an unaffected parent was not sufficient to reclassify it as Likely Benign (LB). Familial testing also showed the two *TELO2* variants to have been inherited from separate parents (*in trans*). Upon reanalysis in 2023, there were case reports several affected individuals harboring the c.392G>A, p.(Gly131Asp) variant in homozygosity or in *trans* with a second *TELO2* variant,[9,10] DECIPHER[11] (IDs: 290986, 402567, 414901). With the addition of further

information from the literature, application of the PM3 criterion and the clinician's confirmation of concordance of the participant's phenotype of Yoo-Hoover-Fong syndrome, the *TELO2* c.392G>A variant was reclassified as LP and the final case-level classification was probable positive.

15846862CSER: a 1-year-old female enrolled with global developmental delay, hypotonia, oculomotor apraxis and molar tooth sign on MRI. Variants were filtered using the PanelApp Cerebellar Hypoplasia, Neurodevelopmental Disorders, and Ophthalmological Disorders gene lists (a PanelApp gene list specific to Joubert syndrome was not available). No reportable variants were identified. A manual literature search for genes linked to autosomal recessive Joubert syndrome was conducted, which identified a reported association with *B9D1*.[12,13] Variants in this gene were analyzed separately, as it was not included on any of the selected PanelApp lists at the time of analysis. The participant was heterozygous for *B9D1* NM_015681.6:c.341G>A NP_056496.1:p.(Arg114Gln) and this variant was reported as LP for Joubert syndrome 27 [MIM: 617120]. This variant is predicted to change the last nucleotide of exon 4 (NM_015681.6) and had been previously reported in a patient with a clinical diagnosis of Joubert syndrome.[13] Splicing studies on that previously-reported patient's blood showed out-of-frame skipping of exon 4.[13] However, this participant's overall case-level classification was inconclusive due to insufficient zygosity.

SUPPLEMENTAL FILE WEB RESOURCES

Online Mendelian Inheritance in Man (OMIM): https://omim.org)

SUPPLEMENTAL FILE REFERENCES

1. Poirier, K., Hubert, L., Viot, G., Rio, M., Billuart, P., Besmond, C., and Bienvenu, T. (2017). *CSNK2B* splice site mutations in patients cause intellectual disability with or without myoclonic epilepsy. Human Mutation *38*, 932–941. https://doi.org/10.1002/humu.23270.

2. Sakaguchi, Y., Uehara, T., Suzuki, H., Kosaki, K., and Takenouchi, T. (2017). Truncating mutation in *CSNK2B* and myoclonic epilepsy. Human Mutation *38*, 1611–1612. https://doi.org/10.1002/humu.23307.

3. Nakashima, M., Tohyama, J., Nakagawa, E., Watanabe, Y., Siew, C.G., Kwong, C.S., Yamoto, K., Hiraide, T., Fukuda, T., Kaname, T., et al. (2019). Identification of de novo CSNK2A1 and CSNK2B variants in cases of global developmental delay with seizures. J Hum Genet *64*, 313–322. https://doi.org/10.1038/s10038-018-0559-z.

4. Ernst, M.E., Baugh, E.H., Thomas, A., Bier, L., Lippa, N., Stong, N., Mulhern, M.S., Kushary, S., Akman, C.I., Heinzen, E.L., et al. (2021). *CSNK2B* : A broad spectrum of neurodevelopmental disability and epilepsy severity. Epilepsia *62*. https://doi.org/10.1111/epi.16931.

5. Yang, Q., Zhang, Q., Yi, S., Qin, Z., Shen, F., Ou, S., Luo, J., and He, S. (2022). De Novo CSNK2B Mutations in Five Cases of Poirier–Bienvenu Neurodevelopmental Syndrome. Front. Neurol. *13*, 811092. https://doi.org/10.3389/fneur.2022.811092.

6. Rehm, H.L., Berg, J.S., Brooks, L.D., Bustamante, C.D., Evans, J.P., Landrum, M.J., Ledbetter, D.H., Maglott, D.R., Martin, C.L., Nussbaum, R.L., et al. (2015). ClinGen — The Clinical Genome Resource. N Engl J Med *372*, 2235–2242. https://doi.org/10.1056/NEJMsr1406261.

7. Goschzik, T., Gessi, M., Denkhaus, D., and Pietsch, T. (2014). *PTEN* Mutations and Activation of the PI3K/Akt/mTOR Signaling Pathway in Papillary Tumors of the Pineal Region. J Neuropathol Exp Neurol *73*, 747–751. https://doi.org/10.1097/NEN.0000000000000093.

8. Mighell, T.L., Evans-Dutson, S., and O'Roak, B.J. (2018). A Saturation Mutagenesis Approach to Understanding PTEN Lipid Phosphatase Activity and Genotype-Phenotype Relationships. The American Journal of Human Genetics *102*, 943–955. https://doi.org/10.1016/j.ajhg.2018.03.018.

9. Albokhari, D., Pritchard, A.B., Beil, A., Muss, C., Bupp, C., Grange, D.K., Delplancq, G., Heeley, J., Zuteck, M., Morrow, M.M., et al. (2023). *TELO2* -related syndrome (YOU-HOOVER-FONG syndrome): Description of 14 new affected individuals and review of the literature. American J of Med Genetics Pt A *191*, 1261–1272. https://doi.org/10.1002/ajmg.a.63142.

10. Zhao, Y., Han, Y., Li, N., Fu, W., Luo, G., Tan, Y., and Qian, X. (2023). Novel compound heterozygous mutations in *TELO2* in an infant with You-Hoover-Fong syndrome: A case report and literature review. Open Life Sciences *18*, 20220602. https://doi.org/10.1515/biol-2022-0602.

11. Firth, H.V., Richards, S.M., Bevan, A.P., Clayton, S., Corpas, M., Rajan, D., Vooren, S.V., Moreau, Y., Pettett, R.M., and Carter, N.P. (2009). DECIPHER: Database of Chromosomal Imbalance and Phenotype in Humans Using Ensembl Resources. The American Journal of Human Genetics *84*, 524–533. https://doi.org/10.1016/j.ajhg.2009.03.010.

12. Romani, M., Micalizzi, A., Kraoua, I., Dotti, M.T., Cavallin, M., Sztriha, L., Ruta, R., Mancini, F., Mazza, T., Castellana, S., et al. (2014). Mutations in B9D1 and MKS1 cause mild Joubert syndrome: expanding the genetic overlap with the lethal ciliopathy Meckel syndrome. Orphanet J Rare Dis *9*, 72. https://doi.org/10.1186/1750-1172-9-72.

13. Katiyar, D., Anderson, N., Bommireddipalli, S., Bournazos, A., Cooper, S., and Goel, H. (2020). Two novel B9D1 variants causing Joubert syndrome: Utility of mRNA and splicing studies. European Journal of Medical Genetics *63*, 104000. https://doi.org/10.1016/j.ejmg.2020.104000.
